# Knowledge, attitude, and practice towards occupational burnout among doctors and nurses in intensive care unit

**DOI:** 10.3389/fpubh.2025.1480052

**Published:** 2025-02-17

**Authors:** Xiahui Lu, Dawei Li, Hu Luo, Lishan Wang, Yan Lou, Yanyan Yu

**Affiliations:** ^1^Department of Critical Care Medicine, Zhejiang Medical & Health Group Hangzhou Hospital, Hangzhou, China; ^2^Department of Critical Care Medicine, The Sixth Medical Center of the PLA General Hospital, Beijing, China; ^3^Department of Respiratory and Critical Care Medicine, First Affiliated Hospital of Army Medical University, Chongqing, China

**Keywords:** ICU, healthcare worker, occupational burnout, knowledge, attitude, practice

## Abstract

**Background:**

Occupational burnout is prevalent among doctors and nurses. This study aimed to investigate the knowledge, attitude, and practice (KAP) of ICU doctors and nurses regarding occupational burnout.

**Methods:**

A cross-sectional study was conducted between December 2023 and June 2024 at the Zhejiang Medical & Health Group Hangzhou Hospital in Zhejiang Province. Demographic information and KAP scores were collected through distributed questionnaires. Occupational burnout was measured by the Maslach Burnout Inventory-General Survey (MBI-GS).

**Results:**

This study included 105 doctors and 165 nurses, with an average age of 32.23 ± 7.38 years. Among all the participants, 6 (2.22%) reported no occupational burnout, 230 (85.19%) experienced moderate occupational burnout, and 34 (12.59%) reported severe occupational burnout. The mean knowledge, attitude, and practice scores were 9.64 ± 4.21 (possible range: 0–18), 29.01 ± 3.15 (possible range: 7–35), and 16.96 ± 4.29 (possible range: 6–30), respectively. Multivariate logistic regression revealed that a higher knowledge score was independently associated with more proactive practice (OR = 1.33, 95% CI: [1.18, 1.50], *p* < 0.001). Structural equation modeling showed that knowledge positively influenced both (*β* = 0.33, *p* < 0.001) and practice (β = 0.37, *p* < 0.001), while practice negatively impacted the MBI-GS (β = −0.92, *p* < 0.001).

**Conclusion:**

Most ICU doctors and nurses exhibited moderate occupational burnout, with insufficient knowledge, positive attitude, and moderate practice toward occupational burnout. Implementing strategies to increase knowledge and promote active practical engagement is essential to effectively mitigate occupational burnout among ICU staff.

## Background

Burnout is defined by emotional exhaustion, depersonalization, and a diminished sense of personal accomplishment, phenomena increasingly prevalent among healthcare professionals worldwide (1). Notably, occupational burnout prevalence rises to 51% among medical and surgical residents (2) and peaks at 80% in physicians (3). Nurses also experience significant rates, ranging from 15 to 60% (4). In addition to compromising personal health, occupational burnout adversely impacts healthcare workers’ perceived competence, and medical performance, and increases the likelihood of medical errors (5, 6). Staff in intensive care units (ICUs)—including registered nurses, medical equipment technicians, and physicians—encounter particularly severe challenges. These roles demand higher performance and understanding, placing intense pressure on ICU personnel (7, 8).

The stress is compounded by a disproportionate ratio of ICU patients to the limited number of available staff, leading to significant psychological strain and deteriorating mental health among healthcare workers (9). Factors contributing to this include high workload, inadequate organizational support, insufficient rewards, workplace violence, and heavy emotional loads, all of which heighten the risk of anxiety, depression, sleep disorders, and burnout syndrome (10, 11). Consequently, regular screening for occupational burnout among ICU clinicians is crucial to safeguard their well-being (12).

The Knowledge, Attitude, and Practice (KAP) survey serves as a diagnostic tool, shedding light on a group’s understanding, beliefs, and behaviors regarding a specific subject, especially in the context of health literacy. This model is based on the idea that knowledge enhances attitude, which in turn, shapes behaviors (13–15). Furthermore, the sequence of the KAP model plays a crucial role in modifying the practice patterns of physicians (16). ICU healthcare professionals are subject to particularly severe stressors due to the demanding nature of their work environments, which include excessive workloads and substantial emotional burdens. Despite existing data from other healthcare populations (17, 18), there is still a lack of research specifically exploring the KAP of occupational burnout among ICU doctors and nurses in China. By understanding the specific experiences and responses of this group to occupational burnout, targeted interventions can be developed to enhance their well-being, improve patient care, and reduce medical errors. This study aims to investigate the KAP of ICU doctors and nurses concerning occupational burnout.

## Methods

### Study design and participants

This cross-sectional study was conducted on ICU doctors and nurses from December 2023 to June 2024 at Zhejiang Medical & Health Group Hangzhou Hospital, Zhejiang Province. Ethical approval for this study was granted by the Medical Ethics Committee of Zhejiang Medical & Health Group Hangzhou Hospital (Approval number: 202311270950000578476). Informed consent was obtained from all study participants. Inclusion criteria: (1) ICU medical doctors and nurses, including those from the Emergency Intensive Care Unit (EICU), General Intensive Care Unit (GICU), Cardiac Intensive Care Unit (CICU), Central Intensive Care Unit (CICU), Respiratory Intensive Care Unit (RICU), Gastrointestinal Intensive Care Unit (GICU), and Oncology Intensive Care Unit (OICU); (2) Doctors and nurses who have been on continuous duty for at least six months at the time of participating in the questionnaire survey; (3) Only doctors and nurses are included. Exclusion criteria: (1) Interns and rotating personnel; (2) Personnel who perform only administrative tasks in the ICU and do not participate in clinical or emergency rescue duties.

### Questionnaire introduction

The questionnaire design was informed by existing literature (19–21). Following the completion of the initial draft, feedback was sought from two seasoned experts: a psychologist with 15 years of experience and an intensivist with 10 years. Based on their suggestions, the questionnaire was refined by adjusting inappropriate descriptions and modifying the response options for several demographic questions. A preliminary survey was administered to 46 participants, resulting in an overall Cronbach’s *α* coefficient of 0.866, which indicates good internal consistency.

The final questionnaire, presented in Chinese, encompasses five sections: demographic information [including age, gender, education level, marital status, family status, monthly income, occupation, years of work experience, average weekly working hours, engagement in teaching and research tasks, job satisfaction rating, PHQ-9 depression screening (22), and GAD-7 anxiety self-assessment (23)], knowledge, attitude, practice, and the Maslach Burnout Inventory-General Survey (MBI-GS) (24, 25). Body mass index (BMI) was calculated as BMI = weight (kg)/height (m)^2^. The knowledge section comprises 9 items, scored from 0 to 18, with responses scored as 2 for ‘very familiar’, 1 for ‘heard of it’, and 0 for ‘unclear’. The attitude section includes 7 questions on a five-point Likert scale, scoring from 7 to 35, where points are allocated from 5 (‘strongly agree’) to 1 (‘strongly disagree’). The practice section contains 6 questions, scored from 6 to 30, where points are assigned from 1 (‘never’) to 5 (‘always’). Scores above 70% of the maximum in each dimension are considered indicative of sufficient knowledge, positive attitude, and proactive practice (26).

The MBI-GS comprises 15 items. The scoring is calculated as follows: Total burnout score = 0.4 × average emotional exhaustion score + 0.3 × average cynicism score + 0.3 × average personal accomplishment score. Total scores within the ranges of 0 ~ 1.49, 1.50 ~ 3.49, and 3.50 ~ 6 correspond to no occupational burnout, moderate occupational burnout, and severe occupational burnout, respectively (24). The primary outcome measures of this study were the KAP scores and burnout scores.

### Questionnaire distribution

An online questionnaire was developed using the Sojump website,^1^ and a QR code was generated for data collection via WeChat. Participants scanned the QR code to access and complete the questionnaire. To ensure quality and completeness, each IP address was allowed only one submission, and all items were mandatory. If participants encountered any issues, research group members were available to provide assistance. All participants completed the questionnaire independently, with researchers only available to provide clarification in cases where participants had technical difficulties with the online survey platform. During the response process, research assistants clarified questions to ensure respondents fully understood the questionnaire and the survey’s intent. The research team reviewed all questionnaires for completeness, consistency, and validity.

### Sample size

The minimum required sample size was calculated using the guideline of 10 times the number of KAP items, as recommended by survey sample size estimation methods. Consequently, the minimum sample size was determined to be 220 (27). To account for an anticipated 20% rate of invalid responses, the adjusted minimum sample size was increased to 264.

### Statistical analysis

Data analysis was conducted using Stata 14.0 (Stata Corporation, College Station, TX, United States). The normal distribution of continuous data was checked using the Kolmogorov–Smirnov test. Continuous variables conforming to the normal distribution were described using mean ± standard deviation (SD), and comparisons between groups were performed using t-tests or analysis of variance (ANOVA). Those with a skewed distribution were presented as medians (ranges) and analyzed using the Wilcoxon-Mann–Whitney U-test or the Kruskal-Wallis analysis of variance. Categorical variables were presented as n (%). Pearson correlation analysis was employed to assess the correlations between knowledge, attitude, practice, and burnout scores. Univariate and multivariate logistic regression were performed to explore the risk factors associated with proactive practice, with 70% of the highest possible score used as the cut-off value (26). The variables with *p* < 0.05 in the univariate analyses were included in the multivariate analyses. A structural equation modeling analysis was conducted to test the hypotheses that (H1) knowledge directly affects attitude, (H2) knowledge directly affects practice, (H3) knowledge indirectly affects practice through attitude, and (H4) KAP directly affects occupational burnout (measured by the MBI-GS). Model fit was evaluated using the Root Mean Square Error of Approximation (RMSEA), Standardized Root Mean Square Residual (SRMR), Tucker–Lewis Index (TLI), and Comparative Fit Index (CFI). Two-sided *p*-values <0.05 were considered statistically significant.

## Results

**Figure 1:**
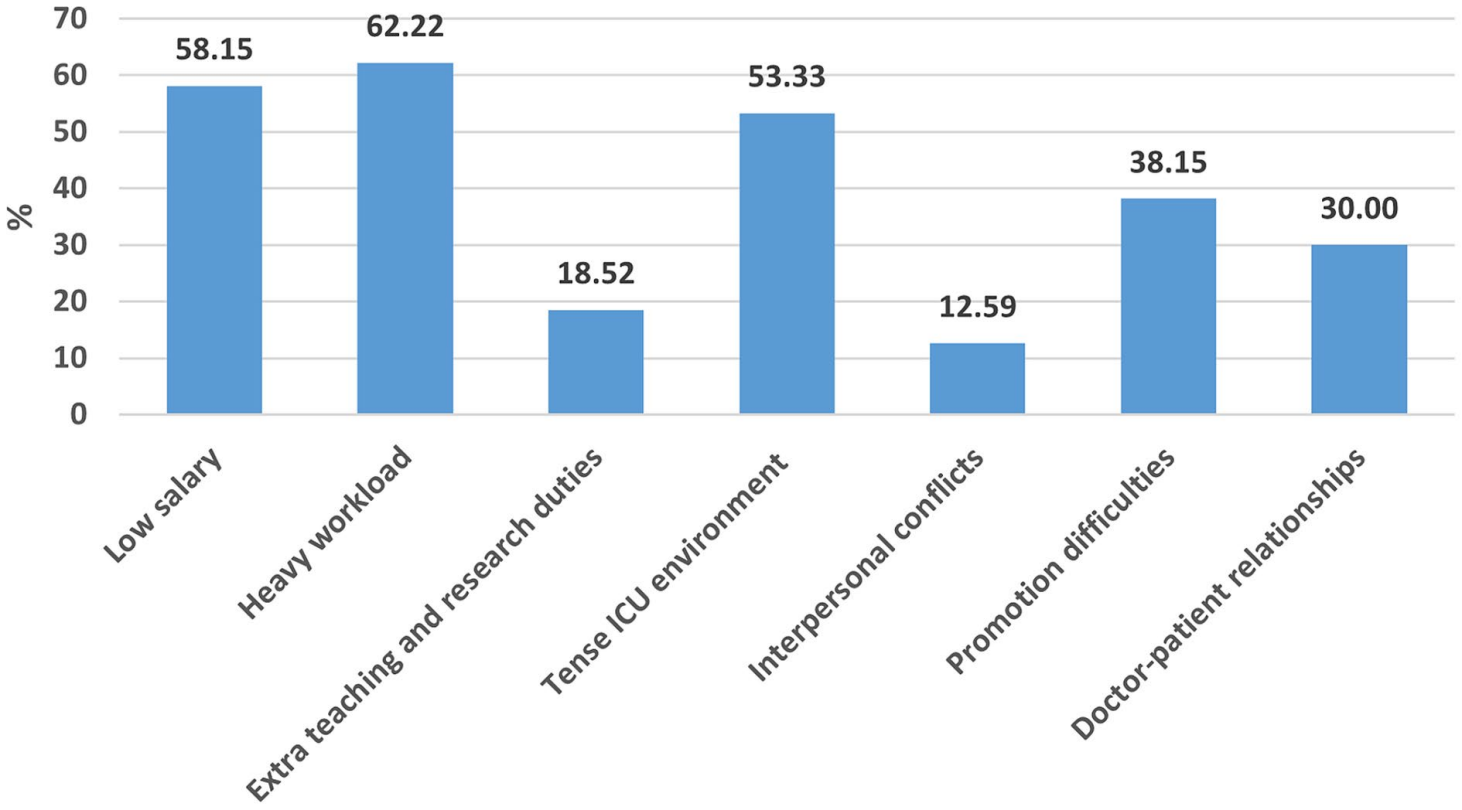
The main issues in the current job of ICU doctors and nurses, including heavy workload, low salary, tense ICU environment, promotion difficulties, doctor-patient relationships, extra teaching and research duties, and interpersonal conflicts, have been identified as key factors contributing to burnout.

A total of 293 questionnaires were collected, with 14 cases excluded due to invalid responses, resulting in 270 valid cases and a validity rate of 92.15%. Among them, 179 (66.30%) were female. The average age of the participants was 32.23 ± 7.38 years. Among the participants, 154 (57.04%) maintained a BMI within the normal range, 154 (57.04%) held a Bachelor’s Degree, 157 (58.15%) were married, 134 (49.63%) had children, and 165 (61.11%) were nurses. Financially, 93 (34.44%) reported a monthly income of 7,000–9,999 yuan. A significant proportion, 115 (42.59%), had 3–10 years of work experience, 134 (49.63%) worked 41–48 h weekly, 218 (80.74%) worked night shifts, 121 (44.81%) undertook teaching tasks, and 76 (28.15%) engaged in scientific research tasks. Additionally, 154 (57.04%) regularly exercised, and 108 (40%) experienced frequent sleep disorder symptoms. Also, 117 (43.33%) scored 5–9 on the depression scale, and 102 (37.78%) exhibited mild anxiety. Among all the participants, 6 (2.22%) reported no occupational burnout, 230 (85.19%) experienced moderate occupational burnout, and 34 (12.59%) reported severe occupational burnout (Tables 1–3). The three primary job-related issues identified were excessive workload, insufficient salary, and a stressful environment in the ICU (Figure 1).

**Table 1:** Demographics characteristic of ICU doctors and nurses.

N = 270	N (%)	Knowledge score		Attitude score		Practice score		Burnout score	
		Mean ± SD	*P*	Mean ± SD	*P*	Mean ± SD	*P*	Mean ± SD	*P*
**Total score**		9.64 ± 4.21		29.01 ± 3.15		16.96 ± 4.29		2.73 ± 0.73	
**Gender**			0.239		0.642		0.613		0.788
Male	91 (33.70)	10.01 ± 4.42		29.15 ± 3.16		17.02 ± 3.92		2.700 ± 0.64	
Female	179 (66.30)	9.45 ± 4.10		28.93 ± 3.14		16.92 ± 4.47		2.751 ± 0.76	
**Age (years)**	32.23 ± 7.38								
**BMI**	22.39 ± 3.29		0.578		0.049		0.411		0.019
Lean	31 (11.48)	9.64 ± 4.66		27.87 ± 3.72		16.70 ± 4.04		2.413 ± 0.53	
Normal	154 (57.04)	9.48 ± 4.18		28.86 ± 3.03		17.28 ± 4.62		2.746 ± 0.73	
Overweight	71 (26.3)	10.19 ± 4.14		29.61 ± 3.06		16.67 ± 3.59		2.836 ± 0.73	
Obesity	14 (5.19)	8.57 ± 3.99		30 ± 2.68		15.28 ± 4.15		2.786 ± 0.74	
**Education**			0.138		0.111		0.031		0.011
Junior College	43 (15.93)	8.46 ± 4.88		27.97 ± 3.75		17.62 ± 5.19		2.588 ± 0.63	
Bachelor’s degree	175 (64.81)	9.73 ± 3.96		29.22 ± 2.93		17.25 ± 4.04		2.674 ± 0.67	
Master’s degree or above	52 (19.26)	10.30 ± 4.32		29.11 ± 3.17		15.38 ± 3.98		3.054 ± 0.85	
**Marital status**			0.002		0.001		0.753		0.389
Unmarried/Divorced/Widowed	113 (41.85)	8.82 ± 4.37		28.24 ± 3.03		17.22 ± 4.59		2.774 ± 0.76	
Married	157 (58.15)	10.23 ± 4.00		29.55 ± 3.12		16.76 ± 4.06		2.705 ± 0.69	
**Any children**			0.006		0.003		0.595		0.297
No	136 (50.37)	9.09 ± 4.31		28.46 ± 3.06		17.27 ± 4.77		2.765 ± 0.79	
Yes	134 (49.63)	10.2 ± 4.05		29.55 ± 3.13		16.62 ± 3.72		2.702 ± 0.64	

**Table 2:** Work-related elements of ICU doctors and nurses.

N = 270	*N* (%)	Knowledge score		Attitude score		Practice score		Burnout score	
		Mean ± SD	*P*	Mean ± SD	*P*	Mean ± SD	*P*	Mean ± SD	*P*
**Job position**			0.020		0.006		0.002		<0.001
Doctor	105 (38.89)	10.26 ± 4.05		29.62 ± 3.01		15.80 ± 3.58		2.973 ± 0.76	
Nurse	165 (61.11)	9.24 ± 4.27		28.61 ± 3.16		17.68 ± 4.54		2.581 ± 0.65	
**Monthly income (yuan)**			0.151		0.015		0.359		0.245
<7,000	70 (25.93)	9.77 ± 4.07		28.58 ± 3.36		17.71 ± 4.92		2.627 ± 0.74	
7,000–9,999	93 (34.44)	8.84 ± 4.19		28.50 ± 3.01		16.84 ± 4.16		2.720 ± 0.72	
10,000–19,999	89 (32.96)	10.24 ± 4.13		29.92 ± 2.89		16.77 ± 4.05		2.871 ± 0.73	
>20,000	18 (6.67)	10.27 ± 4.90		28.72 ± 3.37		15.44 ± 2.97		2.542 ± 0.50	
**Years of working experience**			<0.001		<0.001		0.223		0.062
≤2 years	51 (18.89)	9.56 ± 4.67		28.49 ± 3.23		18.11 ± 4.94		2.882 ± 0.82	
3–10 years	115 (42.59)	8.57 ± 4.14		28.25 ± 3.02		16.95 ± 4.27		2.611 ± 0.68	
11–20 years	75 (27.78)	10.62 ± 3.11		30.08 ± 2.86		16.33 ± 3.88		2.822 ± 0.72	
≥21 years	29 (10.74)	11.48 ± 5.01		30.13 ± 3.20		16.51 ± 3.88		2.729 ± 0.66	
**Weekly working hours**			0.057		0.016		<0.001		<0.001
≤40	38 (14.07)	9.02 ± 5.11		27.63 ± 3.29		18.81 ± 4.41		2.429 ± 0.69	
41–48	134 (49.63)	9.21 ± 4.12		29 ± 3.15		17.42 ± 4.33		2.626 ± 0.64	
49–56	56 (20.74)	10.07 ± 3.33		29.39 ± 2.76		16.46 ± 3.76		2.820 ± 0.57	
≥57	42 (15.56)	11 ± 4.44		29.76 ± 3.16		14.42 ± 3.53		3.237 ± 0.91	
**Work night shifts**			0.002		0.013		0.243		0.872
Yes	218 (80.74)	9.36 ± 4.10		28.82 ± 3.03		16.83 ± 4.40		2.736 ± 0.73	
No	52 (19.26)	10.80 ± 4.51		29.78 ± 3.49		17.44 ± 3.74		2.726 ± 0.66	
**The average number of night shifts per month**			0.012		0.147		0.114		0.083
≤4	65 (24.07)	10.58 ± 4.43		29.4 ± 3.43		17.76 ± 3.84		2.67 ± 0.62	
4–10	92 (34.07)	9.48 ± 4.46		28.58 ± 3.09		16.92 ± 4.73		2.644 ± 0.78	
≥11	113 (41.85)	9.23 ± 3.81		29.12 ± 2.99		16.51 ± 4.11		2.844 ± 0.72	
**Teaching tasks**			0.008		0.005		0.629		0.784
Yes	121 (44.81)	10.41 ± 4.24		29.55 ± 3.15		16.83 ± 4.25		2.729 ± 0.69	
No	149 (55.19)	9.02 ± 4.09		28.56 ± 3.07		17.05 ± 4.33		2.738 ± 0.74	
**Scientific research tasks**			0.004		0.014		0.871		0.066
Yes	76 (28.15)	10.78 ± 3.50		29.75 ± 2.77		16.89 ± 3.75		2.895 ± 0.76	
No	194 (71.85)	9.19 ± 4.38		28.71 ± 3.24		16.97 ± 4.49		2.671 ± 0.69	

**Table 3:** Lifestyle and psychological factors of ICU doctors and nurses.

*N* = 270	*N* (%)	Knowledge score		Attitude score		Practice score		Burnout score	
		Mean ± SD	*P*	Mean ± SD	*P*	Mean ± SD	*P*	Mean ± SD	*P*
**Exercising habit**			0.002		0.059		0.001		0.121
Yes	154 (57.04)	10.33 ± 4.22		29.34 ± 3.22		17.75 ± 4.34		2.655 ± 0.63	
No	116 (42.96)	8.73 ± 4.04		28.56 ± 2.98		15.88 ± 3.99		2.838 ± 0.82	
**Weekly exercise time**			0.085		0.717		0.212		0.677
1 h or less than 1 h	70 (25.93)	11.08 ± 4.13		29.7 ± 3.13		18.45 ± 4.88		2.678 ± 0.70	
1–3 h	62 (22.96)	9.48 ± 4.29		29.16 ± 3.03		17.09 ± 3.87		2.616 ± 0.57	
3–5 h	31(11.48)	10.41 ± 3.65		29.41 ± 3.14		17.25 ± 4.18		2.611 ± 0.57	
Over 5 h	9 (3.33)	9.11 ± 5.41		28.66 ± 5.29		16.44 ± 3.71		2.934 ± 0.76	
**Sleep disorders**			0.759		0.145		0.001		<0.001
Frequent	108 (40)	9.70 ± 4.32		29.39 ± 2.94		16.40 ± 4.61		2.953 ± 0.82	
Occasionally	112 (41.48)	9.59 ± 4.08		28.74 ± 3.01		16.63 ± 3.90		2.643 ± 0.57	
None	50 (18.52)	9.62 ± 4.34		28.76 ± 3.77		18.86 ± 3.93		2.463 ± 0.66	
**Weekly sleep time (hours)**			0.218		0.442		0.013		0.051
≤42	74 (27.41)	10.39 ± 4.24		29.33 ± 3.05		17.02 ± 5.39		2.734 ± 0.79	
43–49	135 (50)	9.31 ± 4.25		28.95 ± 3.05		16.45 ± 3.89		2.805 ± 0.70	
≥50	61 (22.59)	9.45 ± 4.03		28.72 ± 3.45		17.98 ± 3.41		2.576 ± 0.67	
**Your satisfaction rating for the current job is**	6.79 ± 1.96								
**Depression score**			0.360		0.076		0.001		<0.001
0–4	91 (33.7)	9.62 ± 4.45		28.50 ± 3.61		18.08 ± 4.45		2.519 ± 0.63	
5–9	117 (43.33)	9.34 ± 4.08		28.98 ± 2.80		16.70 ± 3.86		2.613 ± 0.53	
10–14	45 (16.67)	10.33 ± 3.93		29.91 ± 2.86		16.33 ± 4.51		3.017 ± 0.77	
15–19	17 (6.3)	10 ± 4.65		29.47 ± 3.04		14.23 ± 4.07		3.963 ± 0.83	
20–27	/								
**Anxiety rating**			0.630		0.085		<0.001		<0.001
None 0–4	137 (50.74)	9.49 ± 4.49		28.64 ± 3.33		17.78 ± 4.35		2.514 ± 0.61	
Mild 5–9	102 (37.78)	9.61 ± 3.77		29.19 ± 2.93		16.35 ± 4.04		2.778 ± 0.62	
Moderate 10–14	17 (6.3)	10.29 ± 4.08		30.29 ± 2.54		17.17 ± 4.11		3.136 ± 0.58	
Major 15–21	14(5.19)	10.5 ± 4.87		29.57 ± 3.03		13 ± 2.77		4.068 ± 0.91	
**Burnout score**			0.007		0.048		0.002		
No burnout (0–1.49)	6 (2.22)	16 ± 3.52		32.66 ± 3.61		23.66 ± 7.68			
Moderate burnout (1.5–3.49)	230 (85.19)	9.50 ± 4.16		28.89 ± 3.19		17.04 ± 4.04			
Severe burnout (3.5–6)	34 (12.59)	9.47 ± 3.88		29.14 ± 2.28		15.17 ± 4.00			

The mean knowledge, attitude, practice, and occupational burnout scores were 9.64 ± 4.21 (possible range: 0–18), 29.01 ± 3.15 (possible range: 7–35), 16.96 ± 4.29 (possible range: 6–30), 2.73 ± 0.73, respectively. Detailed analysis of the KAP dimensions revealed notable gaps. For instance, doctors and nurses demonstrated limited awareness of evidence-based stress reduction strategies (knowledge gap), a tendency to undervalue self-care in managing burnout (attitude misconception), and infrequent application of coping mechanisms in daily practice (practice gap). These deficiencies indicate critical areas for intervention. Meanwhile, their attitude scores were more likely to vary depending on marital status (*p* = 0.001), childbirth status (*p* = 0.003), job position (*p* = 0.006), monthly income (*p* = 0.015), years of working experience (*p* < 0.001), weekly working hours (*p* = 0.016), work night shifts (*p* = 0.013), teaching task (*p* = 0.005), research tasks (*p* = 0.014), and occupational burnout score (*p* = 0.048). Further, their practice scores were more likely to vary depending on education (*p* = 0.031), job position (*p* = 0.002), weekly working hours (*p* < 0.001), habit of exercising (*p* = 0.001), symptoms of sleep disorders (*p* = 0.001), weekly sleep time (*p* = 0.013), depression score (*p* = 0.001), anxiety rating (*p* < 0.001), and occupational burnout score (*p* = 0.002). Moreover, their occupational burnout scores were more likely to vary depending on education (*p* = 0.001), childbirth status (*p* = 0.021), weekly working hours (*p* = 0.002), work night shifts (*p* = 0.049), average number of night shifts (*p* = 0.037), habit of exercising (*p* = 0.002), symptoms of sleep disorders (*p* < 0.001), weekly sleep time (*p* = 0.013), depression score (*p* < 0.001), and anxiety rating (*p* < 0.001) (Tables 1–3).

The responses to the knowledge dimension highlighted notable gaps among ICU healthcare professionals. Only 13.7% were “very familiar” with the definition of occupational burnout (K1), and 17.41% understood its mental health implications, such as anxiety and depression (K3). Additionally, knowledge about physical health consequences (e.g., musculoskeletal disorders, type 2 diabetes) and potential solutions (e.g., work-life balance) was limited (K5 and K9), with only 15.56 and 18.52%, respectively, being “very familiar” with these topics (Table 4). Regarding attitudes, the majority agreed or strongly agreed that burnout is common (84.07%) and a bad sign (85.19%) (A1 and A2). However, a significant proportion of participants underestimated their own capacity for addressing burnout, as 50.37% held neutral or negative views on the potential for self-regulation to alleviate burnout (A5) (Table 5). The responses in the practice dimension revealed low engagement in proactive burnout prevention strategies. Only 6.3% had attended training on burnout prevention (P1), and 15.19% proactively sought psychological support (P5). Furthermore, while 49.63% reported regularly monitoring their work status and emotions (P2), fewer than 35% engaged in effective environmental or lifestyle adjustments, such as regular exercise or improving work-life balance (P3) (Table 6).

**Table 4:** Knowledge dimension distribution.

**Items**	Very familiar, *n* (%)	Heard of it, *n* (%)	Unsure, *n* (%)
Occupational burnout is a syndrome caused by long-term work stress that cannot be effectively controlled.	37 (13.7)	186 (68.89)	47 (17.41)
Occupational burnout manifests as constant feelings of exhaustion or depletion, negativity or apathy towards work, and a sense of detachment, leading to decreased work efficiency.	40 (14.81)	194 (71.85)	36 (13.33)
Occupational burnout is closely associated with adverse mental health effects such as anxiety and depression.	47 (17.41)	184 (68.15)	39 (14.44)
Occupational burnout is related to physical health problems such as insomnia, anxiety, musculoskeletal disorders, and type 2 diabetes.	42 (15.56)	172 (63.7)	56 (20.74)
Healthcare workers are a high-risk group for occupational burnout.	70 (25.93)	169 (62.59)	31 (11.48)
Long working hours are the most common cause of occupational burnout.	72 (26.67)	171 (63.33)	27 (10)
Low salary, lack of autonomy at work, and an uncomfortable work environment can all lead to occupational burnout.	84 (31.11)	158 (58.52)	28 (10.37)
Occupational burnout can significantly impact workers’ social and personal lives.	72 (26.67)	168 (62.22)	30 (11.11)
Occupational burnout can be improved by adjusting work-life balance or mindset.	50 (18.52)	174 (64.44)	46 (17.04)

**Table 5:** Attitude dimension distribution.

Items	Strongly agree, *n* (%)	Agree, *n* (%)	Neutral, *n* (%)	Disagree, *n* (%)	Strongly disagree, *n* (%)
I believe burnout is a very common problem.	94 (34.81)	133 (49.26)	38 (14.07)	5 (1.85)	/
I believe burnout is a very bad sign.	108 (40)	122 (45.19)	35 (12.96)	5 (1.85)	/
I am concerned about the health problems caused by occupational burnout.	129 (47.78)	116 (42.96)	20 (7.41)	5 (1.85)	/
I believe that ICU healthcare workers are more likely to experience occupational burnout compared to other departments.	125 (46.3)	106 (39.26)	32 (11.85)	6 (2.22)	1 (0.37)
I believe occupational burnout is an emotion that can be improved through self-regulation.	32 (11.85)	102 (37.78)	85 (31.48)	41 (15.19)	10 (3.7)
I believe that hospitals should take measures to reduce occupational burnout among ICU healthcare workers.	148 (54.81)	109 (40.37)	13 (4.81)	/	/
I am willing to learn more about occupational burnout.	70 (25.93)	154 (57.04)	40 (14.81)	4 (1.48)	2 (0.74)

**Table 6:** Practice dimension distribution.

Items	Always, *n* (%)	Often, *n* (%)	Sometimes, *n* (%)	Rarely, *n* (%)	Never, *n* (%)
I have attended training on burnout prevention.	3 (1.11)	14 (5.19)	41 (15.19)	100 (37.04)	112 (41.48)
I pay attention to my work status and emotions.	36 (13.33)	98 (36.3)	104 (38.52)	28 (10.37)	4 (1.48)
I will take measures in my daily life to improve the working environment and reduce work stress, such as regular work and rest and strengthening exercise.	28 (10.37)	64 (23.7)	112 (41.48)	58 (21.48)	8 (2.96)
After work, I have a fulfilling spare time life.	20 (7.41)	61 (22.59)	132 (48.89)	57 (21.11)	/
I will proactively seek psychological support and counseling to deal with burnout.	10 (3.7)	31 (11.48)	67 (24.81)	89 (32.96)	73 (27.04)
When I encounter something upsetting at work, I will actively express my opinions and try my best to change the environment.	15 (5.56)	56 (20.74)	107 (39.63)	80 (29.63)	12 (4.44)

Correlation analysis revealed a positive correlation between knowledge and attitude (r = 0.4017, *p* < 0.001), and between knowledge and practice (r = 0.3118, *p* < 0.001). Additionally, there was a negative correlation between practice and occupational burnout scores (r = −0.3359, *p* < 0.001) (Table 7).

**Table 7:** Correlation analysis.

	Knowledge dimension	Attitude	Practice	Burnout score
Knowledge dimension	1			
Attitude	0.4017 (*P*<0.001)	1		
Practice	0.3118 (*P*<0.001)	0.0495 (*p* = 0.4177)	1	
Burnout score	0.0131 (*p* = 0.8303)	0.1421 (*p* = 0.0195)	−0.2317 (*P*<0.001)	1

Multivariate logistic regression for practice dimension showed that knowledge score (OR = 1.33, 95% CI: [1.18, 1.50], *p* < 0.001) was independently associated with proactive practice (Table 8).

**Table 8:** Univariate and multivariate analysis for practice dimension.

Practice	Univariate analysis		multivariate analysis	
	OR (95%CI)	*P*	OR (95%CI)	*P*
**Knowledge score**	1.25 (1.14,1.37)	<0.001	1.33 (1.18,1.50)	<0.001
**Attitude score**	1.11 (0.99,1.24)	0.062		
Gender				
Male				
Female	1.50 (0.69,3.24)	0.301		
**Age (years old)**	0.94 (0.89,1.00)	0.052		
Body mass index				
Lean				
Normal	1.00 (0.35,2.87)	0.988		
Overweight	0.56 (0.16,1.95)	0.37		
Obesity	0.4 (0.04,3.78)	0.424		
Education				
Junior College				
Bachelor’s degree	0.55 (0.24,1.25)	0.155	0.75 (0.25,2.27)	0.618
Master’s degree or above	0.20 (0.05,0.79)	0.022	0.67 (0.08,5.30)	0.709
Marital status				
Unmarried/Divorced/Widowed				
Married	0.53 (0.26,1.06)	0.074		
Do you already have children?				
No				
Yes	0.47 (0.23,0.97)	0.043	1.15 (0.37,3.55)	0.798
Job position				
Doctor				
Nurse	3.96 (1.59,9.86)	0.003	1.91 (0.45,8.00)	0.374
Monthly income (yuan)				
<7,000				
7,000–9,999	0.65 (0.28,1.48)	0.308		
10,000-19,999	0.50 (0.20,1.22)	0.13		
>20,000	0.23 (0.02,1.92)	0.177		
Years of working experience				
≤2 years				
3–10 years	0.47 (0.20,1.07)	0.074	0.44 (0.14,1.38)	0.16
11–20 years	0.30 (0.11,0.81)	0.019	0.25 (0.05,1.27)	0.096
≥21 years	0.21 (0.04,1.03)	0.056	0.15 (0.01,1.26)	0.082
Weekly working hours				
≤40				
41–48	0.63 (0.26,1.52)	0.306	1.33 (0.41,4.24)	0.626
49–56	0.31 (0.09,1.03)	0.057	0.73 (0.15,3.45)	0.702
≥57	0.16 (0.03,0.80)	0.026	0.44 (0.05,3.57)	0.443
Work night shifts				
Yes				
No	1.13 (0.48,2.65)	0.762		
The average number of night shifts per month				
≤4				
4–10	0.65 (0.28,1.53)	0.332		
≥11	0.47 (0.20,1.11)	0.087		
Teaching tasks?				
Yes				
No	1.28 (0.64,2.59)	0.476		
Scientific research tasks				
Yes				
No	1.87 (0.78,4.46)	0.155		
Exercising habit				
Yes				
No	0.49 (0.23,1.04)	0.063		
Weekly exercise time				
1 h or less than 1 h				
1–3 h	0.49 (0.19,1.25)	0.139		
3–5 h	0.43 (0.13,1.48)	0.185		
Over 5 h				
Sleep disorders				
Frequent				
Occasionally	0.79 (0.34,1.86)	0.599	0.48 (0.16,1.40)	0.181
None	2.84 (1.21,6.62)	0.016	1.66 (0.55,5.01)	0.367
Weekly sleep duration (in hours)				
≤42				
43–49	0.45 (0.20,1.00)	0.051		
≥50	0.68 (0.27,1.68)	0.406		
**Your satisfaction rating for the current job is**	1.40 (1.13,1.73)	0.002	1.26 (0.98,1.63)	0.069
Depression score				
0–4				
5–9	0.31 (0.13,0.70)	0.005	0.42 (0.15,1.15)	0.094
10–14	0.51 (0.19,1.37)	0.185	0.75 (0.20,2.78)	0.671
15–19	0.20 (0.02,1.66)	0.139	0.60 (0.05,7.01)	0.686
Anxiety rating				
0–4				
Mild 5–9	0.48 (0.22,1.06)	0.072		
Moderate 10–14	0.96 (0.25,3.59)	0.952		
Severe 15–21				

**Figure 2:**
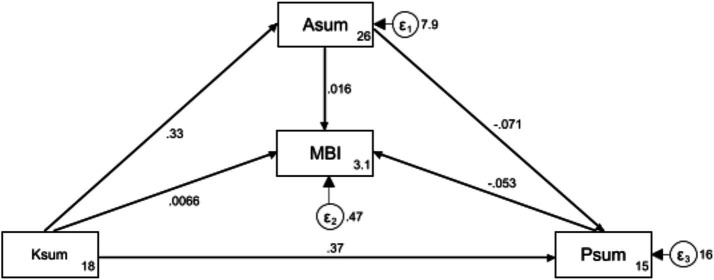
Structural equation modeling illustrating the direct and indirect relationships between knowledge (Ksum), attitude (Asum), practice (Psum), and occupational burnout (measured by MBI). Path coefficients indicate the strength and direction of these relationships, with arrows representing the paths between variables.

Structural equation modeling was conducted to evaluate the relationships between knowledge, attitudes, practices, and occupational burnout (measured by MBI). The fit indices of the structural equation modeling reached the desired range, indicating good model fit results (Supplementary Table S1). The total, direct, and indirect effects of key variables are detailed in Supplementary Table S2. Knowledge directly influenced attitudes (*β* = 0.32, 95% CI: 0.24–0.40, *p* < 0.001). Knowledge significantly affected practice both directly (*β* = 0.36, 95% CI: 0.23–0.49, *p* < 0.001) and indirectly via attitudes (*β* = −0.02, 95% CI: −0.07–0.03, *p* = 0.416). This indicates that knowledge is a critical determinant of both attitudes and practices, reinforcing the importance of targeted educational interventions to improve these dimensions. However, among KAP, only practice showed a direct inverse impact on occupational burnout (*β* = −0.92, 95% CI: −1.20−0.63, *p* < 0.001), underscoring that the more positive and effective the practice is, the lower the burnout score (Supplementary Table S2; Figure 2).

## Discussion

This study reveals that ICU doctors and nurses experience moderate occupational burnout levels, highlighting a disparity between their generally positive attitude towards occupational burnout and their actual knowledge and practice.

In our study, the occupational burnout scores among ICU doctors and nurses revealed a concerning trend: a vast majority, 85.19%, experienced moderate occupational burnout, while 12.59% suffered from severe occupational burnout, and only a minimal 2.22% displayed no signs of occupational burnout. This distribution underscores a significant prevalence of occupational burnout within this cohort, aligning with findings from other studies that also report high levels of occupational burnout among healthcare professionals (28, 29). Factors contributing to these high rates include prolonged working hours, high patient morbidity and mortality, and frequent exposure to critical care stressors. Additionally, personal factors such as younger age, fewer years of professional experience, and inadequate coping mechanisms significantly influence the risk of occupational burnout. For example, a national cross-sectional study in mainland China identified high workload, low physical activity, and insufficient vacation days as key contributors to occupational burnout (28). Another study emphasized that the challenging nature of ICU environments, which include daily encounters with death and ethical dilemmas, further exacerbates stress levels (29). Moreover, factors like personality traits and psychological conditions, such as depression, were highlighted in a study of critical care and emergency nurses in Andalusia, underscoring the complex interplay of individual and systemic factors in occupational burnout prevalence (30).

Notably, the study identifies demographic variables significantly influencing these aspects. For instance, married doctors and nurses and those with children reported higher knowledge and more positive attitude compared to their single, divorced, or widowed counterparts, aligned with previous finding (29). This could be attributed to possibly greater life experience and responsibilities, which might enhance their understanding and coping strategies concerning occupational stress, as familial responsibilities could heighten awareness and adaptive mechanisms against workplace stress (31).

Interestingly, higher educational attainment correlated with increased occupational burnout scores, particularly among those with a Master’s degree or higher. This could reflect a scenario where higher educational levels are associated with greater work expectations and responsibilities, potentially leading to occupational burnout (32). Additionally, doctors scored higher in knowledge yet lower in practice compared to nurses, possibly due to the different nature of their job demands and training, which might emphasize diagnostic and theoretical knowledge over practical coping strategies.

Besides, our study revealed that higher levels of occupational burnout significantly impact the KAP related to occupational burnout among ICU doctors and nurses. Specifically, workers experiencing severe occupational burnout demonstrated lower knowledge and practice scores compared to those with partial or no occupational burnout. This suggests that as occupational burnout increases, the capacity to engage with and apply knowledge effectively diminishes, potentially due to cognitive overload or emotional exhaustion. Extensive weekly work hours were associated with higher knowledge scores yet also the highest occupational burnout scores, which could be a result of increased awareness due to direct exposure to stressors yet a concurrent inability to implement effective coping mechanisms due to time constraints. During the pandemic has revealed that longer working hours correlate with significantly elevated occupational burnout and stress levels (33–35). Although extended hours may offer increased experience and knowledge, they paradoxically heighten the risk of occupational burnout through sustained exposure to stress. Physical exercise emerged as a significant protective factor, associated with better knowledge, improved practice, and lower occupational burnout scores. This aligns with the recommendations by Abdullah S et al., who suggest that physical activity and mindfulness training can be effective interventions for addressing occupational burnout (36). A systematic review involving 11,500 medical students from 13 countries demonstrates an association between physical activity and reduced burnout, as well as improved quality of life among medical students (37). Besides, yoga appears to be effective in the management of stress in doctors and nurses (38).

The results from our multivariate logistic regression, correlation analyses, and structural equation modeling collectively highlight the interconnected nature of KAP in influencing occupational burnout among ICU doctors and nurses. The logistic regression analysis suggests that enhanced knowledge facilitates more proactive practice, a finding echoed in broader healthcare literature, which consistently shows that better educational grounding leads to improved workplace behaviors (39). Similarly, correlation analyses in our study demonstrate a positive relationship between increased knowledge and both improved attitude and practice toward managing occupational burnout, underscoring a trend observed in other study where enhanced understanding directly affects both the emotional and practical responses to workplace stress (40). Further, the structural equation modeling results provide a nuanced view, illustrating that knowledge not only impacts attitude directly but also mediates the relationship between practice and occupational burnout, suggesting that knowledge serves as a crucial lever in reducing occupational burnout through behavioral changes. These analyses together reinforce the notion that comprehensive, knowledge-based interventions are vital. They should aim not only at increasing awareness but also at actively changing how doctors and nurses perceive and respond to stress, thereby fostering a more resilient workforce.

By utilizing the KAP framework, this study identifies specific knowledge gaps, particularly its psychological and physiological effects. Despite being healthcare providers, many still lack full awareness of burnout’s severe outcomes, highlighting the need for targeted educational interventions. While most professionals recognize burnout as common and harmful, many underestimate their ability to manage it. This gap between awareness and self-efficacy should be addressed to improve burnout prevention. Additionally, proactive burnout prevention practices are underutilized. Only 6.3% of participants received burnout prevention training, and just 5.19% sought psychological support. These findings suggest a lack of standardized approaches to managing burnout, even in hospitals with mental health services.

To effectively address the prevalent issue of occupational burnout among ICU doctors and nurses in China, a multifaceted strategy that leverages both technology and organizational support is essential. Developing an online education platform utilizing popular social media channels such as WeChat could provide accessible, engaging content that enhances understanding and management of occupational burnout (41, 42). This platform could feature interactive modules and real-time feedback mechanisms that allow workers to assess their occupational burnout levels and receive personalized coping strategies. Additionally, incorporating mental health support into existing digital communication tools could offer a confidential space for workers to seek professional advice and peer support. Organizational efforts should also focus on optimizing the work environment to reduce unnecessary stressors (43, 44). This could involve streamlining administrative processes and creating dedicated relaxation spaces within the hospital, equipped with amenities such as massage chairs and soothing music. In addition, providing gym or yoga studio memberships to healthcare workers as part of employee benefits could encourage active exercise habits, better managing stress and occupational burnout. Moreover, providing career development opportunities and educational subsidies can further empower healthcare workers by enhancing their professional skills and job satisfaction.

This study has several limitations. First, its cross-sectional design prevents the determination of causal relationships between variables. Additionally, this design captures data at a single point in time, which may not reflect the dynamic nature of occupational burnout that evolves over time due to individual, organizational, or external factors affecting ICU practice. Second, the study’s reliance on self-reported data may introduce bias, as participants might overestimate their knowledge or underreport their levels of occupational burnout. Third, while cross-sectional studies, including ours, inherently limit the ability to evaluate the effectiveness of interventions, this research provides unique insights into occupational burnout from the perspective of the KAP framework, which can serve as a basis for future intervention studies. Fourth, while the multiple data points have enabled us to identify possible links between variables, these findings may be closely tied to factors such as cultural norms or support networks, which require further exploration through qualitative methods. As the survey was conducted using electronic questionnaires, we were unable to determine the total number of individuals who were invited to participate. Therefore, the response rate could not be accurately assessed. Another limitation of this study is the potential response bias, as those experiencing burnout may be more likely to participate, potentially leading to overreporting of burnout and underreporting of related knowledge and practices. Finally, the focus on a single hospital limits the generalizability of the findings to other settings or regions. Despite these limitations, this study’s strengths include a robust sample size and the use of validated instruments like the MBI-GS, which enhance the reliability of the findings. Additionally, the comprehensive analysis techniques, including correlation analysis, multivariate logistic regression, and structural equation modeling, provide a deep understanding of the factors influencing occupational burnout among ICU doctors and nurses. By applying the KAP framework, this study offers a unique perspective, addressing one aspect of burnout and laying the groundwork for targeted interventions. Future research should incorporate larger sample sizes and employ longitudinal study designs to capture the evolving nature of occupational burnout over time. Additionally, combining these approaches with qualitative methods could provide deeper insights into contextual factors such as cultural norms, organizational dynamics, and individual coping mechanisms, enhancing the understanding of occupational burnout among ICU doctors and nurses.

## Conclusion

In conclusion, the majority of ICU doctors and nurses exhibit moderate levels of occupational burnout, with inadequate knowledge but a generally positive attitude toward managing occupational burnout. This study underscores the direct and indirect impact of knowledge on practice and occupational burnout levels. Given the clear linkage between knowledge and proactive practice, it is crucial for healthcare institutions to enhance educational and training programs focused on occupational burnout prevention and management strategies among ICU workers.

## Data Availability

The original contributions presented in the study are included in the article/Supplementary material, further inquiries can be directed to the corresponding author.

## Footnotes

^1^ https://www.wjx.cn/
