# Supplementary material for: Knowledge, attitude, and practice towards occupational burnout among doctors and nurses in intensive care unit
Source: Front Public Health. 2025 Feb 17;13:1480052. doi: 10.3389/fpubh.2025.1480052 (PMC11872883; doi:10.3389/fpubh.2025.1480052)
Supplement: Supplementary file 1 [file Table_1.docx]

**Table S1. Path model fitting**

| **Indicators** | **Reference** | **Results** |
| --- | --- | --- |
| **RMSEA** | <0.08 Good | 0.000 |
| **SRMR** | <0.08 Good | 0.000 |
| **TLI** | >0.8 Good | 1.000 |
| **CFI** | >0.8 Good | 1.000 |
