# Supplementary material for: Knowledge, attitude, and practice towards occupational burnout among doctors and nurses in intensive care unit
Source: Front Public Health. 2025 Feb 17;13:1480052. doi: 10.3389/fpubh.2025.1480052 (PMC11872883; doi:10.3389/fpubh.2025.1480052)
Supplement: Supplementary file 2 [file Table_2.docx]

**Table S2. Analysis of direct and indirect effects**

| **Model paths** |  | **Total effects** | | **Direct Effect** | | **Indirect effect** | |
| --- | --- | --- | --- | --- | --- | --- | --- |
|  |  | β (95% CI) | P | β (95% CI) | P | β (95% CI) | P |
| **Asum <-** |  |  |  |  |  |  |  |
|  | Ksum | 0.32 (0.24,0.40) | <0.001 | 0.32 (0.24,0.40) | <0.001 | — | — |
| **MBI <-** |  |  |  |  |  |  |  |
|  | Asum | 0.28 (-0.15,0.72) | 0.207 | 0.21 (-0.19,0.63) | 0.298 | 0.06 (-0.09,0.22) | 0.414 |
|  | Psum | -0.92 (-1.20, -0.63) | <0.001 | -0.92 (-1.20, -0.63) | <0.001 | — | — |
|  | Ksum | -0.0 (-0.30,0.29) | 0.974 | 0.23 (-0.08,0.56) | 0.152 | -0.24 (-0.44, -0.04) | 0.019 |
| **Psum <-** |  |  |  |  |  |  |  |
|  | Asum | -0.07 (-0.24,0.09) | 0.414 | -0.07 (-0.24,0.09) | 0.414 | — | — |
|  | Ksum | 0.34 (0.22,0.45) | <0.001 | 0.36 (0.23,0.49) | <0.001 | -0.02 (-0.07,0.03) | 0.416 |
